# mRNA expression of CDH3, IGF2BP3, and BIRC5 in biliary brush cytology specimens is a useful adjunctive tool of cytology for the diagnosis of malignant biliary stricture

**DOI:** 10.1097/MD.0000000000004132

**Published:** 2016-07-08

**Authors:** Tae Ho Kim, Jae Hyuck Chang, Hee Jin Lee, Jean A Kim, Yeon Soo Lim, Chang Whan Kim, Sok Won Han

**Affiliations:** a Department of Internal Medicine; b Institute of Clinical Medicine Research; c Department of Hospital Pathology; d Department of Radiology, College of Medicine, The Catholic University of Korea, Seoul, Republic of Korea.

**Keywords:** brush cytology, cholangiocarcinoma, endoscopic retrograde cholangiopancreatography, messenger RNA, polymerase chain reaction

## Abstract

Although advances have been made in diagnostic tools, the distinction between malignant and benign biliary strictures still remains challenging. Intraductal brush cytology is a convenient and safe method that is used for the diagnosis of biliary stricture, but, low sensitivity limits its usefulness. This study aimed to demonstrate the usefulness of mRNA expression levels of target genes in brush cytology specimens combined with cytology for the diagnosis of malignant biliary stricture. Immunohistochemistry for cadherin 3 (CDH3), p53, insulin-like growth factor II mRNA-binding protein 3 (IGF2BP3), homeobox B7 (HOXB7), and baculoviral inhibitor of apoptosis repeat containing 5 (BIRC5) was performed in 4 benign and 4 malignant bile duct tissues. Through endoscopic or interventional radiologic procedures, brush cytology specimens were prospectively obtained in 21 and 35 paitents with biliary strictures. In the brush cytology specimens, the mRNA expressions levels of 5 genes were determined by real-time polymerase chain reaction. Immunohistochemistry for CDH3, p53, IGF2BP3, HOXB7, and BIRC5 all showed positive staining in malignant tissues in contrast to benign tissues, which were negative. In the brush cytology specimens, the mRNA expression levels of CDH3, IGF2BP3, HOXB7, and BIRC5 were significantly higher in cases of malignant biliary stricture compared with cases of benign stricture (*P* = 0.006, *P* < 0.001, *P* < 0.001, and *P* = 0.001). The receiver-operating characteristic curves of these 4 mRNAs demonstrated that mRNA expression levels are useful for the prediction of malignant biliary stricture (*P* = 0.006, *P* < 0.001, *P* < 0.001, and *P* = 0.002). The sensitivity and specificity, respectively, for malignant biliary stricture were 57.1% and 100% for cytology, 57.1% and 64.3% for CDH3, 76.2% and 100% for IGF2BP3, 71.4% and 57.1% for HOXB7, and 76.2% and 64.3% for BIRC5. When cytology was combined with the mRNA levels of CDH3, IGF2BP3, or BIRC5, the sensitivity for malignant biliary stricture improved to 90.5%. The measurement of the mRNA expression levels of CDH3, IGF2BP3, and BIRC5 by real-time polymerase chain reaction combined with cytology was useful for the differentiation of malignant and benign biliary strictures in brush cytology specimens.

## 1 Introduction

The incidence of bile duct cancer has continued to increase worldwide, and it is particularly common in Asia and the Middle East. Unfortunately, this cancer has a poor prognosis, with a 5-year survival rate of less than 30% even in patients with localized extrahepatic bile duct cancer.^[1]^ Although early detection and complete resection are the only effective curative approaches for this dreadful cancer, sensitive tumor markers to detect this tumor during their early stages still have not been developed, and many patients present with late-stage disease.

Patients with bile duct cancer usually present with biliary stricture, and it is often difficult to differentiate malignant from benign biliary strictures. Many imaging studies including transabdominal or endoscopic ultrasonography, multichannel dynamic computed tomography, magnetic resonance imaging, and positron emission tomography-computed tomography facilitate the diagnosis of malignant biliary strictures. Although these imaging modalities possess various sensitivities, a final pathological diagnosis by biopsy is required to confirm malignancy. Since a bile duct with a tumor is narrow, and the targeting of the tumor with biopsy forceps by fluoroscopic guidance is indirect, a bile duct biopsy is a challenging procedure. Even with a successful biopsy, the size of the biopsy specimen is often insufficient for a proper diagnosis.

Brush cytology through the biliary stricture is a convenient and safe method for pathological diagnosis. However, brush cytology is limited by low sensitivity, although its specificity is high.^[2,3]^ Therefore, adjunctive tools combined with cytology are needed to increase the sensitivity of brush cytology. As for these attempts, some approaches have been investigated using brush cytology specimens such as fluorescence in situ hybridization,^[4,5]^ mRNA expression levels of specific tumor-related genes,^[6–10]^ and DNA analysis.^[11,12]^ Among them, the measurement of target gene mRNA expression levels is a promising adjunctive tool. To determine tumor-specific target genes, a gene expression microarray was used. It revealed many up-regulated genes in primary biliary cancers and biliary cancer-derived cell lines.^[13]^ Fifty-two commonly up-regulated genes associated with bile duct cancers have been detected by a genome-wide analysis, and strong cadherin 3 (CDH3) and baculoviral inhibitor of apoptosis repeat containing 5 (BRIC5), survivin) immunohistochemical staining in cholangiocarcinoma has been observed.^[14]^ Recent studies have found that the mRNA levels of some genes such as S100 calcium-binding protein P (S100P), nimA-related kinase 2 (NEK2), msh Homeobox 2 (MSX2), insulin-like growth factor II mRNA-binding protein 3 (IGF2BP3), and homeobox B7 (HOXB7) increased in brush cytology specimens of malignant biliary stricture.^[6–10]^ It was also reported that the immunohistochemical expression of p53 was effective in the diagnosis of bile duct cancer.^[15,16]^ Thus, additional supporting data are needed to clarify the appropriate target genes and the usefulness of target gene mRNA expression combined with cytology. This study aimed to evaluate whether cytology combined with the mRNA expression of 5 promising tumor-related genes (CDH3, BIRC5, IGF2BP3, HOXB7, and p53) in brush cytology specimens is useful for the differentiation of malignant and benign biliary strictures.

## 2 Methods

### 2.1 Patients

Patients with benign or malignant biliary strictures who were consecutively admitted to our institute from April 2013 to May 2014 and who consented to participate in the study were prospectively included. After a sufficient sample size was obtained, the recruitment of patients was stopped. The patients were followed up over 1 year until May 2015. The target sensitivity of mRNA expression was 80%, at which rate the sensitivity of the combination of cytology and mRNA expression was over 90%. The conditions were as follows: α = 0.05, power 80%, sensitivity of mRNA expression in benign tissue = 30%, and ratio of cases of malignant-to-benign strictures = 2:1. The minimum required sample sizes of malignant and benign patients were 21 and 11, respectively (32 total). Malignant biliary stricture was diagnosed based on the results of brush cytology, endoscopic retrograde forcep biopsy, percutaneous forcep biopsy, or surgical biopsy. Benign biliary stricture was diagnosed when the initial examination, which included the pathological evaluation, was negative for malignancy, and when no change in the lesion was demonstrated after more than 1 year. The exclusion criteria were as follows: young age (<20 years), no consent given, and malignant biliary stricture due to conditions other than bile duct cancer such as pancreatic cancer, stomach cancer, hepatocellular carcinoma, or metastatic lymph nodes.

### 2.2 Ethics

All participants provided their written informed consent to participate in this study. This study was approved by Bucheon St. Mary's Hospital Institutional Research Board (HC12TISI0135) and was conducted in complete compliance with the Declaration of Helsinki.

### 2.3 Immunohistochemistry and interpretation

Surgical specimens from 8 patients who underwent surgery were embedded in paraffin and were diagnosed as benign (4) or malignant (4); these specimens were subjected to immunohistochemistry (IHC). IHC was performed on 3-μm thick paraffin sections using an automated immunohistochemical stainer (Ventana Medical Systems, Inc., Tucson, AZ) according to the manufacturer's protocol. The sections were deparaffinized using EZ Prep solution (Ventana). Deparaffinized tissue sections were pretreated with cell-conditioning solution (Ventana) at 95°C for 60 minutes. To block the endogeneous peroxidase activity, the ultraviolet inhibitor step was performed at 37°C for 4 minutes before the detection of the primary antibody. The primary antibodies were diluted in Dako antibody diluent (Dako Cytomation, Glostrup, Denmark) with background-reducing components and were used at the following dilutions: p53 (1:100 dilution, Dako), HOXB7 (1:100 dilution, Abcam Ltd., Cambridge, UK), IGF2BP3 (1:50 dilution, Abcam Ltd.), CDH3 (prediluted, Abcam Ltd.), and BIRC5 (1:250 dilution, Abcam Ltd.). The sections were incubated with primary antibodies at room temperature for 32 minutes, and staining was then detected using the EnVision Plus system (Dako, Carpinteria, CA); this is a ready-to-use, peroxidase-based dual-link kit that detects primary mouse and rabbit antibodies. The reaction was developed with diaminobenzidine (DAB; Dako) for 5 minutes, and the slides were counterstained with hematoxylin II (Ventana) for 4 minutes and bluing reagent (Ventana) for 4 minutes. Negative control sections were treated in the same manner, but they were incubated with antibody diluent instead of the primary antibody.

The immunohistochemical staining was interpreted independently by JAK and THK, who were blinded as to the results. The expression of each antibody was quantified based on the extent of staining. The expression was considered positive if distinct immunoreactivity was observed in one-third or more of the tumor cells with a more than mild staining intensity.

### 2.4 Brush cytology

All patients underwent endoscopic retrograde cholangiopancreatography (ERCP) to evaluate the biliary strictures, and brush cytology was attempted during ERCP. If brush cytology failed during the ERCP, percutaneous transhepatic cholangiography (PTC) was performed. In those cases, brush cytology was performed 1 or 2 days later via the tract used for PTC. A standard cytology brush catheter (Cytomax II double lumen cytology brush, Cook Endoscopy, Winston-Salem, NC) was used for the brush cytology under fluoroscopic guidance. The brush and its sheath were inserted into the bile duct over a guidewire and were positioned near the stricture site. The brush was advanced from the sheath and was moved back and forth across the biliary stricture site 10 times. Then, the brush was withdrawn into the catheter, and the catheter with the brush was retrieved. The retrieved brush was soaked and shaken in saline to remove the specimens from the brush. The saline specimen mixture was immediately submitted to the laboratory for mRNA expression analysis. Then, the brush catheter was re-inserted for the conventional cytology work-up. Brush cytology was performed again using the same maneuver as the first brush procedure. After retrieval of the brush catheter, the brush portion of the brush catheter that contained the specimens was cut, soaked in CytoRich Red Preservative Fluid, and then sent to the pathology department. There, the specimens were separated from the brush, and the fluid that contained the cells was centrifuged for 5 minutes at 1500 rpm. Tris-buffered saline was added to the precipitate, and the mixture was re-centrifuged. The cytology specimen slides were produced automatically using the precipitates. After the brush cytology, biopsies with forceps were performed when possible.

### 2.5 Cytological evaluation

The pathologist (JAK) who was blinded to the mRNA results evaluated the brush cytology slides. The cytological results were classified as follows: malignant, suspicious for malignancy, atypia, and benign. In the present study, “malignant” and “suspicious for malignancy” were considered positive for malignancy.

### 2.6 RNA extraction and reverse transcription

The brush specimens in the saline were centrifuged for 10 minutes at 2000 rpm at 4°C, and 5 volumes of RNA Protect Cell Reagent (Qiagen, Valencia, CA) were added to one volume of cell pellet. Then, the cells were mixed by shaking, pipetting, or vortexing, and were stored at –70°C before RNA purification, which was performed using the RNeasy Plus Micro Kit (Qiagen) according to the manufacturer's protocol. Specifically, 1 μg of purified total RNA was reverse transcribed into first-strand cDNA using a Quantitect Reverse Transcription Kit (Qiagen), which includes a genomic DNA elimination step.

### 2.7 Real-time polymerase chain reaction

Real-time polymerase chain reaction (RT-PCR) amplification and relative quantification of CDH3, p53, IGF2BP3, HOXB7, BIRC5, and β-actin mRNAs were performed using TaqMan gene expression assays (Table 1, Applied Biosystems, Foster City, CA) in a LightCycler 480 PCR system (Roche, Manheim, Germany). All assays used similar amplification efficiency, and a ΔCT experimental design was used for relative quantification. The reactions were performed in a 20-μL volume in duplicates using a TaqMan Probe Master Mix (Roche), and 10 ng of cDNA was used in each reaction. β-actin served as an endogenous control. A no-template control was included in each quantitative RT-PCR experiment to confirm the absence of DNA contamination in the reagents used for the amplification. The results were analyzed by LightCycler 480 software 1.2 (Roche).

**Table 1:**
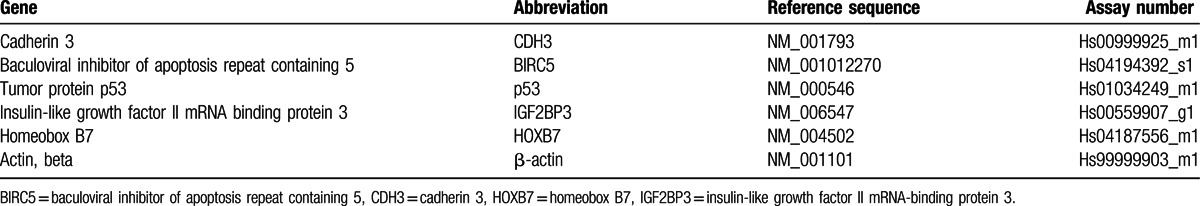
Gene expression assays used for real-time polymerase chain reaction.

### 2.8 Statistical analysis

For quantitative variables, the mean ± standard deviation (SD) was given. Quantitative variables were analyzed by a Mann–Whitney *U* test or Pearson chi-square test. Receiver-operating characteristic (ROC) curves were plotted, and the areas under the ROC curves (AUC) were calculated to determine the diagnostic ability of the mRNA expression of each gene. The cut-off mRNA expression values were determined using the ROC curves. The sensitivity, specificity, positive predictive value, negative predictive value, and 95% confidence intervals (CIs) were calculated for each individual diagnostic method and combined diagnostic methods. *P* values that were less than 0.05 were considered significant. Statistical analyses were performed with SPSS, version 20 (SPSS, Inc., Chicago, IL).

## 3 Results

### 3.1 Patient characteristics

In all, 38 patients were initially enrolled; 2 patients with malignant biliary stricture and 1 patient with benign biliary stricture were excluded due to inappropriate specimens. All cytology procedures were safely performed without unwanted harm to the patients. Finally, 35 patients were enrolled in the study, and their detailed characteristics are described in Table 2. The malignant biliary strictures consisted of common bile duct carcinoma, Klatskin tumor, and gallbladder carcinoma. Four patients with gallbladder cancer exhibited direct invasion into the liver and bile duct, which resulted in bile duct stricture and jaundice. The benign strictures consisted of choledocholithiasis, postsurgical complications, inflammatory strictures, and idiopathic strictures. The mean age of the patients was 69.6 ± 12.1 years, and the total number of male patients was 16 (46%). Brush cytology was performed by the ERCP method in 24 patients (69%) and by the PTC method in 11 patients (31%).

**Table 2:**
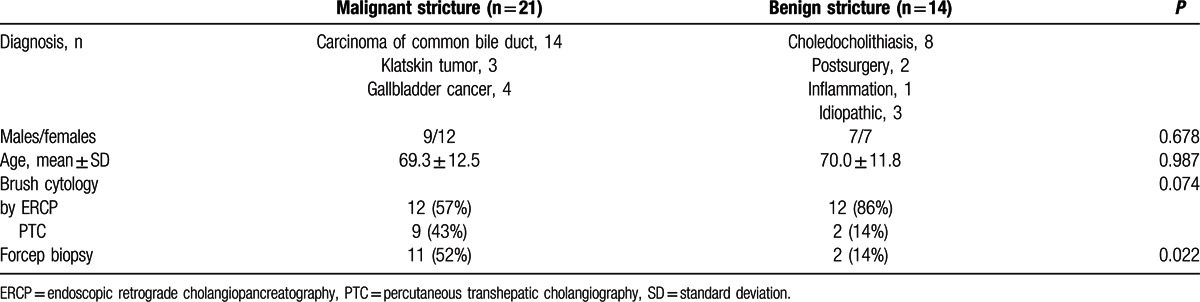
Patient characteristics.

### 3.2 Immunohistochemistry

**Figure 1:**
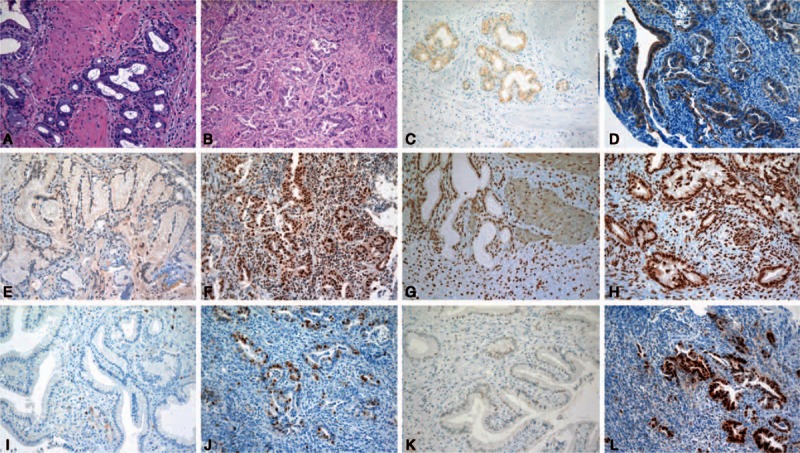
Hematoxylin–eosin and immunohistochemical stains of benign and malignant bile duct tissues (×200). A, B, Hematoxylin–eosin staining in benign and malignant tissues; C, D, CDH3 staining in benign and malignant tissues; E, F, IGF2BP3 staining in benign and malignant tissues; G, H, HOXB7 staining in benign and malignant tissues; I, J, BIRC5 staining in benign and malignant tissues; K, L, p53 staining in benign and malignant tissues. BIRC5 = baculoviral inhibitor of apoptosis repeat containing 5, CDH3 = cadherin 3, HOXB7 = homeobox B7, IGF2BP3 = insulin-like growth factor II mRNA-binding protein 3.

Immunohistochemistry stains for BIRC5, IGF2BP3, CDH3, HOX7, and p53 showed positive staining in all 4 malignant tissues (Fig. 1). IGF2BP3, CDH3, and HOX7 were strongly expressed in more than half the area in all malignant tissues (Fig. 1D, F, and H). Staining for BIRC5 and p53 revealed moderate to high intensity of expression in one-third to one-half the area (Fig. 1J and L). These 5 genes were not expressed in benign tissues (Fig. 1E, I, and K), except that CDH3 and HOX7 showed mild expression in the epithelium and in epithelial and stromal cells in 1 benign specimen, respectively (Fig. 1C and G).

### 3.3 mRNA expressions in brush cytology specimens

**Figure 2:**
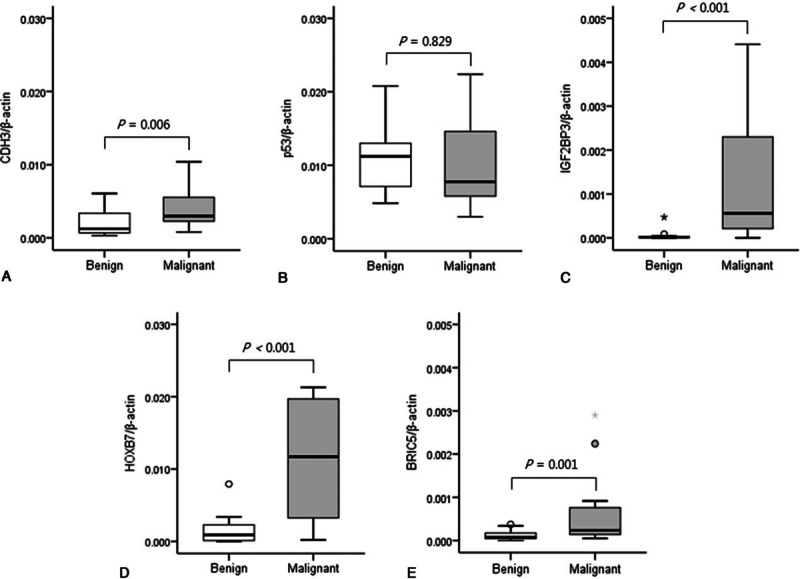
Relative mRNA expression levels in brush cytology specimens from malignant and benign biliary strictures. The mRNA expression levels of CDH3/β-actin (A), p53/β-actin (B), IGF2BP3/β-actin (C), HOXB7/β-actin (D), and BIRC5/β-actin (E) in brush cytology specimens were compared between benign and malignant biliary strictures. The box and whisker plot show the median, interquartile range, and range of values. BIRC5 = baculoviral inhibitor of apoptosis repeat containing 5, CDH3 = cadherin 3, HOXB7 = homeobox B7, IGF2BP3 = insulin-like growth factor II mRNA-binding protein 3.

**Figure 3:**
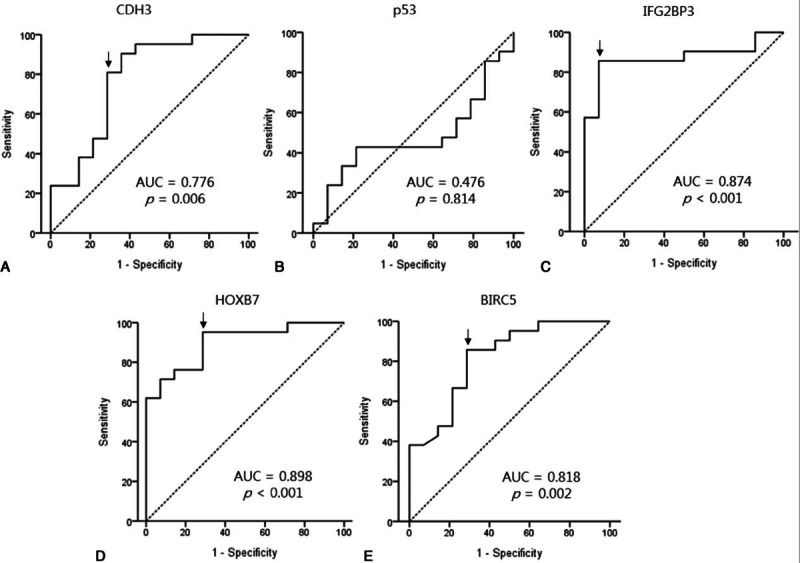
Receiver-operating characteristic (ROC) curve analyses for the relative mRNA expression levels as diagnostic markers of malignancy in the biliary tract. ROC curves for CDH3 (A), p53 (B), IGF2BP3 (C), HOXB7 (D), and BIRC5 (E) were plotted. The area under the ROC curves (AUC) and *P* values are shown in the curves. The cut-off values (arrows) are indicated in each curve. BIRC5 = baculoviral inhibitor of apoptosis repeat containing 5, CDH3 = cadherin 3, HOXB7 = homeobox B7, IGF2BP3 = insulin-like growth factor II mRNA-binding protein 3.

The expression levels of CDH3, IGF2BP3, HOXB7, and BIRC5 mRNA in malignant biliary strictures were significantly higher than in benign strictures (*P* = 0.006, <0.001, <0.001, and 0.001, respectively; Fig. 2). However, the p53 mRNA expression levels did not differ between the benign and malignant biliary strictures. The ROC curves demonstrated that the CDH3, IGF2BP3, HOXB7, and BIRC5 mRNA expression levels were useful for the prediction of malignant biliary stricture (*P* = 0.006, <0.001, <0.001, and 0.002, respectively; Fig. 3). The AUCs were 0.776 (95% CI 0.605–0.946) for CDH3, 0.476 (95% CI 0.280–0.672) for p53, 0.874 (95% CI 0.750–0.998) for IFG2BP3, 0.898 (95% CI 0.796–1.000) for HOXB7, and 0.818 (95% CI 0.674–0.963) for BIRC5. The AUCs of IFG2BP, HOXB7, and BIRC5 were higher than 0.8. The AUC of HOXB7, which exhibited the highest diagnostic ability for malignant biliary stricture, was the highest at 0.898.

### 3.4 Sensitivity and specificity

Using the ROC curves, the cut-off values were determined to be 0.00212 for CDH3 mRNA expression, 0.000999 for IGF2BP3 mRNA expression, 0.00137 for HOXB7 mRNA expression, and 0.000129 for BIRC5 mRNA expression. The respective sensitivities and specificities are described in Table 3. The sensitivities of IGF2BP3 mRNA and BIRC5 mRNA were the highest at 76.2%, whereas the next highest sensitivity was that of HOXB7 mRNA at 71.4%. The lowest sensitivity was for CDH3 mRNA and cytology at 57.1%. When cytology and one of the expression levels of CDH3, IGF2BP3, or BIRC5 mRNA were combined (the specimens were diagnosed as malignant if either of the 2 tests was positive for malignancy), the sensitivity increased to 90.5% (95% CI 68.2–98.3; Table 4). In particular, when the cytology results were combined with IGF2BP3 mRNA expression, the sensitivity and specificity were maximized to 90.5% and 100%, respectively, with an accuracy of 94.3%.

**Table 3:**
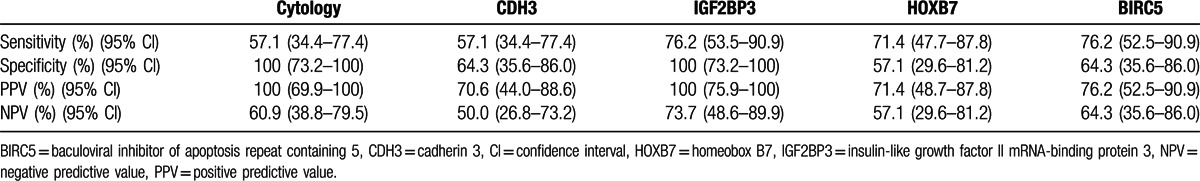
Performance characteristics of cytology and mRNA expressions for malignant biliary stricture.

**Table 4:**
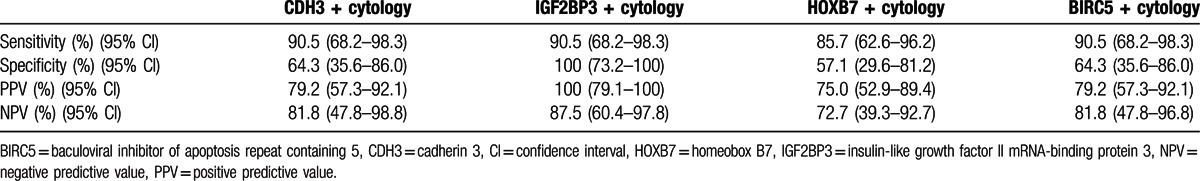
Performance characteristics of combined diagnostic methods for malignant biliary stricture.

## 4 Discussion

This study demonstrated that CDH3, IGF2BP3, HOXB7, and BIRC5 are expressed in malignant bile duct tissues, and that the mRNAs expression levels of these genes in brush cytology specimens combined with cytologic analysis are useful diagnostic markers for malignant biliary stricture.

The differentiation between malignant and benign biliary strictures is crucial to determine the treatment plan in patients with biliary stricture. Since pancreatico-biliary tract surgeries are major operations, and patients who undergo this particular type of surgery exhibit considerable morbidity and mortality, unnecessary major surgery in patients with benign biliary strictures should definitely be avoided. In addition, the confirmation of malignancy before surgery is important so that the patients understand the necessity of the surgery and their subsequent prognosis. Although various imaging modalities facilitate the diagnosis, they are not without flaws, and they each have various and limited sensitivities. Consequently, the pathological diagnosis of biliary strictures is necessary for a confirmative diagnosis. Several ways to obtain the specimens may be attempted. Brush cytology or forcep biopsy during ERCP or PTC, needle biopsy by endoscopic ultrasonography, and biopsy using percutaneous transhepatic choledochoscopy, mother baby scope, Spyglass system, or direct peroral choledochoscopy have been introduced. These methods are promising, but a majority of them are not widely used due to high cost, difficulty with the techniques, and a lack of established results.

Brush cytology is one of the conventional methods that is easy to perform and has low cost and high specificity. However, the utility of brush cytology is limited due to its low sensitivity.^[3]^ In addition to the basic limitation of cytology itself compared with biopsy, brush cytology often yields only a small number of malignant cells due to the growth pattern and nature of bile duct cancers. Therefore, tools that can facilitate a diagnosis based on a small specimen size are needed to improve the conventional brush cytology yield. RT-PCR, which has a high sensitivity, can detect specific mRNA even with a very small number of malignant cells.^[7]^ Therefore, the detection of tumor-associated gene overexpression in biliary tract specimens using PCR may be effective in the diagnosis of bile duct cancer. Several tumor-related genes have been shown to be up-regulated in bile duct cancer based on a microarray analysis,^[13,14]^ and tumor-related gene mRNAs isolated from biliary brush specimens have been demonstrated to be suitable for the molecular analysis of biliary diseases using PCR and microarray.^[17]^ Furthermore, RT-PCR for detection of mRNA is a rapid, inexpensive, and reliable method, and all genes can be processed simultaneously during the same amplification protocol.^[10]^ The mRNA expression levels of several tumor-related genes, including MSX2, human aspartyl (asparaginyl) beta-hydroxlase (HAAH), HOXB7, S100P, IGF2BP3, and NEK2, have been recently studied.^[6–10]^ In the present study, the mRNA levels of HOXB7 and IGF2BP3 were re-evaluated, and the mRNA of CDH3, p53, and BIRC5 were assessed for the first time to determine their efficacy in the diagnosis of bile duct cancer based on brush cytology. The mRNAs expressed in the tumors showed a somewhat wide range of expression. The level of mRNA expression in the tumor might depend on various factors such as tumor differentiation and individual variation.

CDH3 is a calcium-sensitive cell adhesion molecule that plays a role in cell growth and differentiation.^[18,19]^ CDH3 is overexpressed in the majority of pancreatic cancer and in various other malignancies, including bile duct, gastric, and colorectal cancers.^[14,20–22]^ CDH3 overexpression was reported to be associated with an aggressive phenotype and poor prognosis of endometrial carcinomas.^[18]^ In this study, CDH3 mRNA was significantly highly expressed in malignant biliary strictures and was a good predictor of malignant biliary stricture. The sensitivity improved to 90.5% when CDH3 mRNA levels were combined with conventional cytology. IGF2BP3 is an onco-fetal RNA-binding protein that promotes tumor cell proliferation by enhancing IGF-II protein synthesis, and induces cell adhesion and invasion by stabilizing CD44 mRNA.^[23]^ IGF2BP3 expression has been studied in multiple types of human neoplasms such as malignant mesothelioma and biliary cancer.^[8,10,14,24]^ IGF2BP3 has been shown to be a biomarker of enhanced tumor aggressiveness, and its expression has been correlated with increased risk of metastases and decreased survival of patients with esophageal squamous cell carcinoma, bladder cancer, and colon cancer.^[25–27]^ In the present study, IGF2BP3 mRNA was significantly highly expressed in malignant biliary strictures and was a good predictor of malignant biliary stricture. Particularly, the sensitivity and specificity of the diagnostic methods were highly increased to 90.5% and 100%, respectively, if cytology was combined with IGF2BP3 mRNA expression. HOXB7 is a homeobox-containing gene and transcription factor that is normally expressed during development and that is aberrantly overexpressed in ovarian and breast cancer.^[28,29]^ HOXB7 is overexpressed at both the mRNA and protein levels in biliary cancer, but is not detectable in normal biliary epithelium.^[13,14]^ Our study demonstrated that HOXB7 mRNA was significantly highly expressed in malignant biliary stricture and was the best predictor of malignant biliary stricture among the studied genes based on the 0.898 AUC. BIRC5 is expressed during fetal development and is undetectable in terminally differentiated adult tissues. However, BIRC5 is prominently expressed in transformed cell lines and in all of the most common human cancers, including lung, colon, pancreas, prostate, and breast cancer.^[30]^ BIRC5 is involved in the inhibition of apoptosis; therefore, elevated BIRC5 expression may play a crucial role in the development of intrahepatic cholangiocarcinoma.^[14]^ Nuclear BIRC5 expression in cholangiocarcinoma was shown to be associated with poor prognosis.^[31]^ In the current study, BIRC5 mRNA was significantly highly expressed in malignant biliary strictures and was also a good predictor of this condition. The sensitivity improved to 90.5% when the CDH3 mRNA levels were combined with cytology. p53 is a representative tumor suppressor gene. Point mutations in the p53 gene represent one of the most common genetic alterations found in human cancer. High immunohistochemical p53 expression in cholangiocarcinoma has been reported,^[15]^ and p53 immunostaining combined with histology of specimen obtained by biopsy forceps improved the sensitivity of the diagnosis of bile duct cancer.^[16]^ However, other studies reported that p53 immunocytochemistry added no definite additional value to brush cytology in the discrimination between benign and malignant strictures.^[32,33]^ Our study showed that p53 mRNA had no significant role in the differentiation of bile duct cancer based on brush cytology.

In this study, IHC was done in the specimens of 8 patients. Although the IHC demonstrated obvious differences between malignant and benign tissues, the number of IHC stains was not sufficient for a statistical comparison. We wanted to confirm the protein expression of the target genes in a preliminary IHC study before mRNA expression study. Further IHC researches in the future may provide more detailed information such as tissue expression levels of genes or the relationship between gene expressions and tumor differentiation. The power of our study had been set to 80% in the sample size calculation, but the actual power was 74.2% due to slightly lower sensitivity of mRNA expression in malignant strictures than we had expected. Although this power is within an acceptable range, it could limit the application of this study. Hopefully, further study with a larger number of patients will be performed to validate our study.

In conclusion, CDH3, IGF2BP3, and BIRC5 mRNA expression levels as determined by RT-PCR combined with cytology were effective for the differentiation of malignant and benign biliary strictures in brush cytology specimens. Therefore, target gene mRNA in brush cytology is a useful adjunctive diagnostic tool of cytology for the diagnosis of malignant biliary stricture.
